# A Practical Treatise on Fractures and Dislocations

**Published:** 1871-10

**Authors:** 


A Practical Treatise on Fractures and Dislocations. By Frank Hastings Hamilton, A. M., M. D. Fourth edition. Revised and improved. Philadelphia: Henry C. Lea, Publisher.

This standard work of Prof. Hamilton's is, we believe, almost the only complete and exhaustive treatise on the subject in the English language. It is a volume that should be in the possession of every medical practitioner who undertakes to deal with this troublesome branch of surgery. With it at hand for reference, and to fall back upon as authority in case of need, and with the exercise of a moderate degree of care and skill on the part of the physician, most of the troublesome and disagreeable mal-practice suits, to which this class of injuries so frequently gives rise, might be avoided.

No essential alterations appear to have been made since the former editions.

The name of Henry C. Lea as publisher is of itself a sufficient guarantee of the excellence of the mechanical execution of the work. Clear, legible type, good paper, and substantial leather binding being the invariable accompaniments of all volumes received from that house.

